# Gardening can induce pulmonary failure: *Aspergillus* ARDS in an immunocompetent patient, a case report

**DOI:** 10.1186/s12879-014-0600-6

**Published:** 2014-11-26

**Authors:** Nina Jung, Silke Mronga, Susanne Schroth, Timon Vassiliou, Frank Sommer, Eduard Walthers, Christian Aepinus, Andreas Jerrentrup, Claus Vogelmeier, Angelique Holland, Rembert Koczulla

**Affiliations:** Department of Medicine, Pulmonary and Critical Care Medicine, University Medical Center Giessen and Marburg, Philipps-University Marburg, Member of the German Center for Lung Research (DZL), Marburg, Germany; Department of Anesthesiology and Critical Care Medicine, Marburg, Germany; Department Institute for Medical Microbiology and Hygiene, Marburg, Germany; Department of Diagnostic and Interventional Radiology, Marburg, Germany; Department Institute for Virology, Marburg, Germany

**Keywords:** Aspergillus fumigatus, Sepsis, ECMO, Immunocompetent patient, Treatment

## Abstract

**Background:**

Acute *Aspergillus fumigatus* infection in immunocompetent patients is rare. This is the first known case of a patient who survived *Aspergillus* sepsis after being treated early with veno-venous extracorporeal membrane (ECMO) and antifungal therapy.

**Case presentation:**

An immunocompetent 54-year-old woman was exposed to plant mulch during gardening and subsequently developed pulmonary failure that progressed to sepsis with multiorgan failure. Owing to her severe clinical condition, she was treated for acute respiratory distress syndrome (ARDS) with veno-venous ECMO. Empiric antifungal therapy comprising voriconazole was also initiated owing to her history and a previous case report of aspergillosis after plant mulch exposure, though there was no microbiological proof at the time. *A. fumigatus* was later cultured and detected on antibody testing. The patient recovered, and ECMO was discontinued 1 week later. After 7 days of antifungal treatment, *Aspergillus* antibodies were undetectable.

**Conclusions:**

In cases of sepsis that occur after gardening, clinicians should consider *Aspergillus* inhalation as an aetiology, and early antimycotic therapy is recommended.

**Electronic supplementary material:**

The online version of this article (doi:10.1186/s12879-014-0600-6) contains supplementary material, which is available to authorized users.

## Background

Russell et al. hypothesized that a correlation may exist between gardening and serious illness based on the case of a man who developed acute respiratory distress syndrome (ARDS), likely caused by *Aspergillus fumigatus* after spreading rotted tree and plant mulch in his garden [1]. The patient reported being engulfed by clouds of dust from the mulch. The patient died despite receiving extracorporeal membrane oxygenation (ECMO) therapy. We encountered a similar patient at our hospital 10 years ago, who developed illness after spreading decayed tree and plant mulch. This was the background for the presented case.

## Case presentation

A 54-year-old female patient presented to the emergency department of a local hospital reporting cough with respiratory distress. The patient did not smoke or consume alcohol, and had no allergies; however, she reported several years of secondary cigarette smoke exposure from her husband. Auscultation of the lungs revealed a crackling noise. On laboratory examination, the absolute white blood cell count was 12.2 × 10^9^/l, the C-reactive protein (CRP) was 190 mg/l, and the procalcitonine (PCT) was 0.17 μg/l. The chest radiographs showed bilateral lung infiltrates. Therefore, the patient was diagnosed with a community-acquired pneumonia. Her primary physician had started the patient on cefuroxime three days earlier, which was changed to moxifloxacine (400 mg/d) and piperacillin/tazobactame (18 g/d). Because the patient was in respiratory failure, non-invasive ventilation was initiated. After two days of therapy, her respiratory function showed no improvement; therefore, the patient was transferred to our tertiary centre.

The patient had no history of immunosuppressive disease or treatment. Blood tests for HIV, hepatitis, and chronic autoimmune disorders were negative. The laboratory examination was repeated and showed an absolute white blood cell count of 24.0 × 10^9^/l. Neutrophilia and lymphopenia were observed, and the T4:T8 ratio (4.69) was elevated. In addition, the CRP was significantly elevated (341 mg/l); the PCT was 0.4 μg/l, and the erythrocyte sedimentation rate was 70 mm/h. An electrocardiogram and echocardiogram did not show any abnormality.

**Figure 1:**
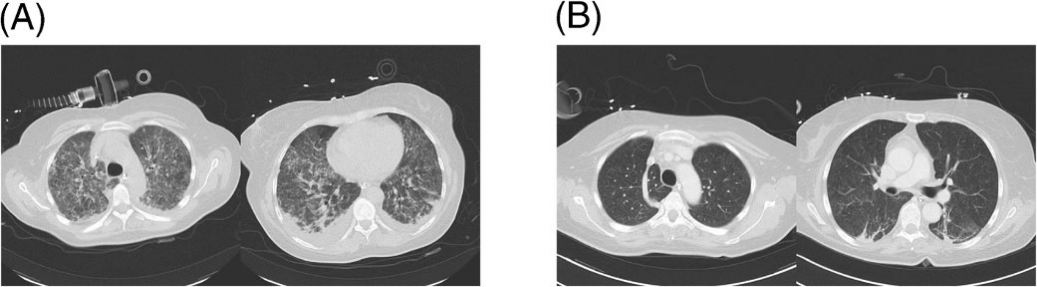
**Initial CT scan (A) with bilateral diffuse interstitial nfiltrate and (B) the partial resolution after starting treatment.**

The respiratory failure was refractory to non-invasive ventilation and required intubation with controlled mechanical ventilation. The initial Horowitz Index was 56 mmHg. Computed tomography (CT) showed bilateral diffuse interstitial infiltrates (Figure 1); therefore, all ARDS criteria were satisfied [2]. Bronchoscopic examination showed generalized mucosal inflammation. Bronchoscopic biopsies were obtained and evaluated by the microbiology department. Broad-spectrum antibiotic therapy was initiated comprising meropenem (3 g/d) and levofloxacine (1 g/d). The initial microbiological tests of the blood samples and the bronchoalveolar lavage fluid (BALF) did not show any bacterial growth.

The cardiovascular function began to destabilize in the patient. Vasoactive support was administered to treat hypotension and comprised norepinephrine (maximum 0.6 μg/kg/min) and dobutamine (maximum 4.6 μg/kg/min); thus, all criteria of septic shock were fulfilled [3]. The gas exchange showed no significant improvement despite treatment (Horowitz Index 77 mmHg). Consequently, veno-venous ECMO was implanted. The ARDS was also treated with intravenous methylprednisolone [4]. Owing to renal failure, continuous veno-venous hemofiltration was initiated.

**Figure 2:**
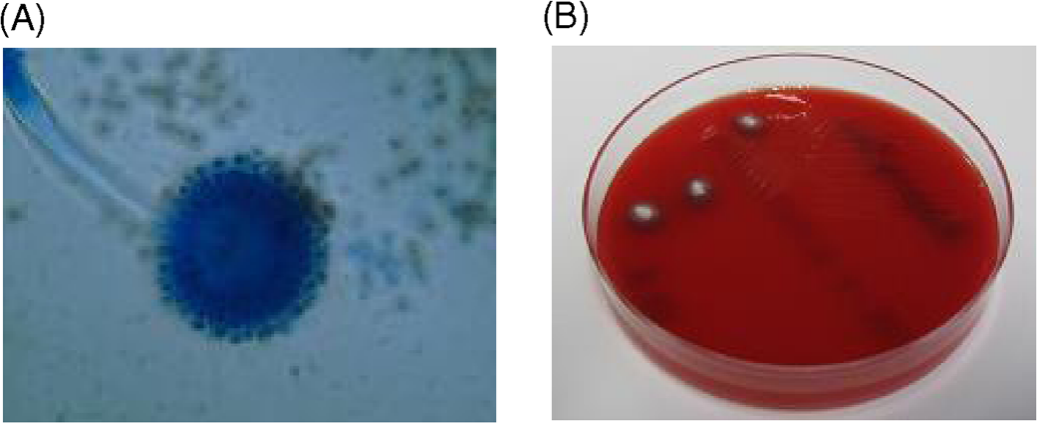
**Microscopy of** ***Aspergillus fumigatus*** **(A) and** ***Aspergillus fumigatus*** **growth on nutrient agar (B).**

The underlying cause of the patient's critical condition could not be determined; therefore, her family was asked once more on any special activities of the patient within the last few days prior to admission. The relatives reported that two days before her symptoms appeared, the patient had been gardening using non-fermented tree bark, which dispersed a large amount of dust. A fungal aetiology was suspected, and we started empirical antifungal treatment with voriconazole (300 mg/d) based on a similar clinical course in a case of *Aspergillus* infection following exposure to non-fermented tree bark [1]. Further laboratory analysis revealed elevated antibody titres for *A. fumigatus* (IgG 255 U/ml and IgM 79 U/ml), and the galactomannan test was positive (*Aspergillus* antigen: 4.6). Microbiological examination of the BALF revealed growth of *A. fumigatus* hyphae (Figure 2). No bacteria were cultured from the BALF.

Because of the increasing azole resistance of *A. fumigatus* across Europe and the critical condition of the patient, we also began caspofungin (50 mg/d) therapy one day after initiating voriconazole. The patient immediately responded to the therapy with dramatic clinical improvement. We reduced the catecholamine support, and ECMO was discontinued on day 7. The patient was successfully weaned from the ventilator two days later. On day 7 of antifungal treatment, *Aspergillus* antigen was no longer detectable.

A repeat CT showed partial resolution with persistent infiltrates and areas of partial consolidation in the basal segments of both lobes. The patient was discharged for rehabilitation after 19 days of hospitalization.

## Discussion

*A. fumigatus* sepsis after exposure to plant mulch during gardening. *Aspergillus* spores are often found in decaying plant matter. Inhalation of these spores can cause allergic broncho-pulmonary aspergillosis, pulmonary aspergilloma, or pulmonary aspergillosis. Sepsis appears to be very rare.

The previously published case [1] and our case did not show immunosuppression, but both patients had epithelial pathology due to smoking, smoke exposure (as in our patient), or recurrent bronchitis. Potentially, these factors may have damaged the epithelium and predisposed the patient to *A. fumigatus* infection or otherwise increased susceptibility. Marihuana smoking is reportedly associated with aspergillosis. Chronic respiratory diseases such as chronic obstructive pulmonary disease (COPD) also increase the susceptibility to aspergillosis [5].

The extreme exposure to *Aspergillus* spores may have caused acute respiratory failure due to an acute hypersensitivity reaction, but the clinical course is extremely unusual. The clinical symptoms began two days after exposure, and discontinuation of mulch exposure did not ameliorate her symptoms. There are no known cases of hypersensitivity pneumonitis requiring mechanical ventilation. The *Aspergillus* antibodies and culture findings are suggestive of sepsis, which responded to antimycotic therapy. We did not observe any evidence of an acute hypersensitivity reaction, which should have responded to steroid treatment.

## Conclusions

In the present case, we suspect that the combination of early empiric antimycotic treatment and early lung replacement therapy by veno-venous ECMO saved the life of our patient. In cases where sepsis occurs after gardening, doctors should consider *Aspergillus* inhalation as a potential cause, and early antimycotic therapy should be recommended.

### Ethical considerations

Written informed consent was obtained from the patient for publication of this case report and any accompanying images. A copy of the written consent is available for review by the Editor of this journal.

## Authors' contributions

SM, SS, AJ, TV cared for the patient in the intensive care unit. EW analysed and interpreted the radiological data. FS and CA performed the microbiological and virological analyses. NJ, CV, AH, and RK took also cared for the patient in the intensive care unit, performed the literature review, and drafted the manuscript. All authors read and approved the final manuscript.

## Authors’ original submitted files for images

Below are the links to the authors’ original submitted files for images. Authors’ original file for figure 1 Authors’ original file for figure 2
