# Supplementary figures and images for: Gardening can induce pulmonary failure: Aspergillus ARDS in an immunocompetent patient, a case report
Source: BMC Infect Dis. 2014 Nov 26;14:600. doi: 10.1186/s12879-014-0600-6 (PMC4253624; doi:10.1186/s12879-014-0600-6)

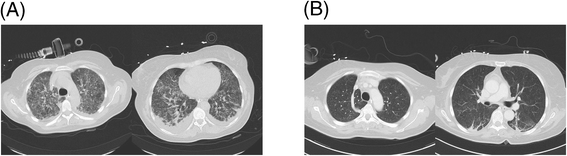

Supplement: Supplementary file 1 — Authors’ original file for figure 1 [file 12879_2014_600_MOESM1_ESM.gif]

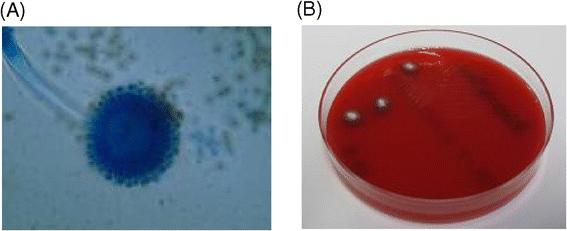

Supplement: Supplementary file 2 — Authors’ original file for figure 2 [file 12879_2014_600_MOESM2_ESM.gif]
